# Functional Outcomes of Retrograde Intramedullary Screw Nailing for Displaced Midshaft Clavicle Fractures in Adults

**DOI:** 10.7759/cureus.102289

**Published:** 2026-01-25

**Authors:** Rahul H Sakhare, Pallav P Agrawal, Sushil Mankar, Darshan Sharma

**Affiliations:** 1 Orthopaedics and Traumatology, NKP Salve Institute of Medical Sciences and Research Centre and Lata Mangeshkar Hospital, Nagpur, IND

**Keywords:** clavicle fractures, intramedullary nails, plating, screw nail, tens nailing

## Abstract

Background

Midshaft clavicle fractures are quite common among clavicle injuries in adults. Although traditionally managed conservatively, displaced fractures often lead to nonunion, malunion, and shoulder dysfunction. While plate fixation offers strong stabilization, it requires a large incision and extensive soft-tissue dissection. Intramedullary fixation has become a minimally invasive alternative, but earlier devices such as Kirschner wires and elastic stable intramedullary nails (ESIN) have shown limitations, including implant migration, irritation, and inadequate rotational stability. This study evaluated the functional outcomes of a newly designed titanium retrograde intramedullary screw nail in the management of displaced midshaft clavicle fractures.

Methods

A prospective study was conducted between July 2023 and July 2025, including 24 adult patients (17 males, 7 females; mean age 35.5 years) with Allman Type 1 displaced midshaft clavicle fractures. Fixation was performed using a titanium intramedullary screw nail inserted via a lateral (retrograde) entry under fluoroscopic guidance. Postoperatively, patients were immobilized in a sling for four weeks and subsequently underwent progressive physiotherapy. Evaluations were conducted at 3, 6, and 12 months postoperatively using clinical, radiological, and functional assessments. Functional outcome was measured using the Disabilities of the Arm, Shoulder and Hand (DASH) score. Operative parameters and complications were also recorded.

Results

The most common mechanism of injury was a road traffic accident (62.5%). The mean operative time was 45.4 minutes, with an average blood loss of 80.8 mL and an incision length of 13.9 mm. The mean hospital stay was 4.9 days. All fractures achieved union, with one case of delayed union in an elderly osteoporotic patient following nail backout. No infections, implant breakage, or neurovascular complications were observed. The mean DASH score improved from 73.5 preoperatively to 18.8 at 12 months (p < 0.001). Nineteen patients (79.2%) had excellent results and five (20.8%) had fair results.

Conclusion

Retrograde intramedullary fixation using a titanium screw nail provides a safe, stable, and minimally invasive method for managing displaced midshaft clavicle fractures. It ensures reliable union, minimal soft-tissue complications, excellent cosmetic outcomes, and early functional recovery. Larger randomized studies are recommended to confirm these encouraging results.

## Introduction

Clavicle fractures account for approximately 2.5%-5% of all fractures in adults, with the majority seen in the midshaft region (69%) and with varying degrees of displacement [1,2]. Traditionally, midshaft clavicle fractures have been managed conservatively; however, this approach often leads to complications such as nonunion, malunion, and shoulder asymmetry [3,4]. A frequent complication of middle third clavicle fractures is malunion, which occurs when fracture healing is associated with angulation and shortening, with an unsatisfactory cosmetic outcome. Studies have shown that malunions can lead to neurological or functional impairments, particularly when the shortening exceeds 2 cm [5].

Recently, early surgical intervention has been advised as an effective method to significantly lower the risk of nonunion and malunion in midshaft clavicle fractures [6]. Open reduction and plate-and-screw fixation are widely regarded as the standard surgical treatment, offering strong fixation and earlier postoperative mobilization [7]. However, this technique requires a larger incision, exposes more soft-tissue structures, and is associated with various postoperative complications [8].

Intramedullary fixation has gained popularity as an alternative treatment due to its minimally invasive nature, reduced periosteal stripping, stress distribution, and ease of operation [9]. Despite these advantages, earlier intramedullary implants such as Kirschner wires have fallen out of favor due to concerns about insufficient stability [10,11]. More advanced options, including elastic stable intramedullary nails (ESIN) and locking clavicle nails, have since been developed and widely utilized. However, ESIN has been associated with complications such as malunion, superficial wound infections, and implant backout. Similarly, locking flexible clavicular nails pose risks, including irritation at the entry site [12,13].

Titanium elastic nails (TEN) using a medial entry are frequently used for treating displaced midshaft clavicle fractures and have demonstrated favorable clinical outcomes as an alternative to conservative management [14]. However, TEN have limitations, including inadequate resistance to rotation and shortening. Additionally, they have been linked to complications such as hardware irritation, medial perforation, lateral penetration, and nail breakage. Reports indicate that up to 44% of patients have experienced issues, including medial migration and clavicular shortening of 5 mm or more [15].

To address these challenges, a new implant, the titanium intramedullary screw nail, was applied in this study to evaluate its clinical effectiveness in managing displaced midshaft clavicle fractures while aiming to minimize postoperative complications. This implant has been used previously in midshaft clavicle fractures in which the nail was inserted through a medial entry. However, several complications such as medial prominence, entry-site infection, and backout were observed. Hence, we developed a new technique with a lateral (retrograde) entry to address the above-mentioned complications associated with other surgical techniques.

## Materials and methods

Study design and setting

This was a prospective clinical study conducted at the Department of Orthopaedics, NKP Salve Institute of Medical Sciences and Research Centre and Lata Mangeshkar Hospital, Nagpur, India, between July 2023 and July 2025, after approval from the Institutional Review Board (IRB No. NKPSIMS & RC and LMH/IEC/3/2023). All participants provided written informed consent prior to inclusion.

Study population 

A total of 24 adult patients (17 males and 7 females; mean age 35.5 years, range 21-58 years) presenting with displaced midshaft clavicle fractures were included. The diagnosis was confirmed by clinical and radiographic evaluation using standard anteroposterior and 15° cephalic tilt clavicle views.

Inclusion criteria

Patients aged 18 years and above; displaced midshaft clavicle fractures (Allman Type I/Robinson Type 2B1 or 2B2); closed fractures with no associated major neurovascular injury; and patients medically fit for surgery and willing for follow-up were included in the study.

Exclusion criteria

Open or pathological fractures; fractures extending into the medial or lateral third of the clavicle; polytrauma patients or those with ipsilateral upper limb injuries; and patients lost to follow-up before final assessment were excluded from the study.

Sample size calculation

The sample size was estimated based on the expected improvement in functional outcome measured by the Disabilities of the Arm, Shoulder and Hand (DASH) score from previous studies on intramedullary fixation of clavicle fractures. Assuming a mean difference of 25 points, SD of 20, power of 80%, and α = 0.05, the minimum required sample size was calculated to be 20. To account for potential dropouts, 24 patients were included [16]. 

Sample size calculation: n = [(Zα/2 + Zβ)² × 2σ²] / Δ²

Ethical approval and patient consent

This study was approved by our Hospital Ethics Committee and conducted in compliance with ethical guidelines. Written informed consent was obtained from all participants prior to their inclusion in the study.

Characteristics of the intramedullary screw nail

**Figure 1:**
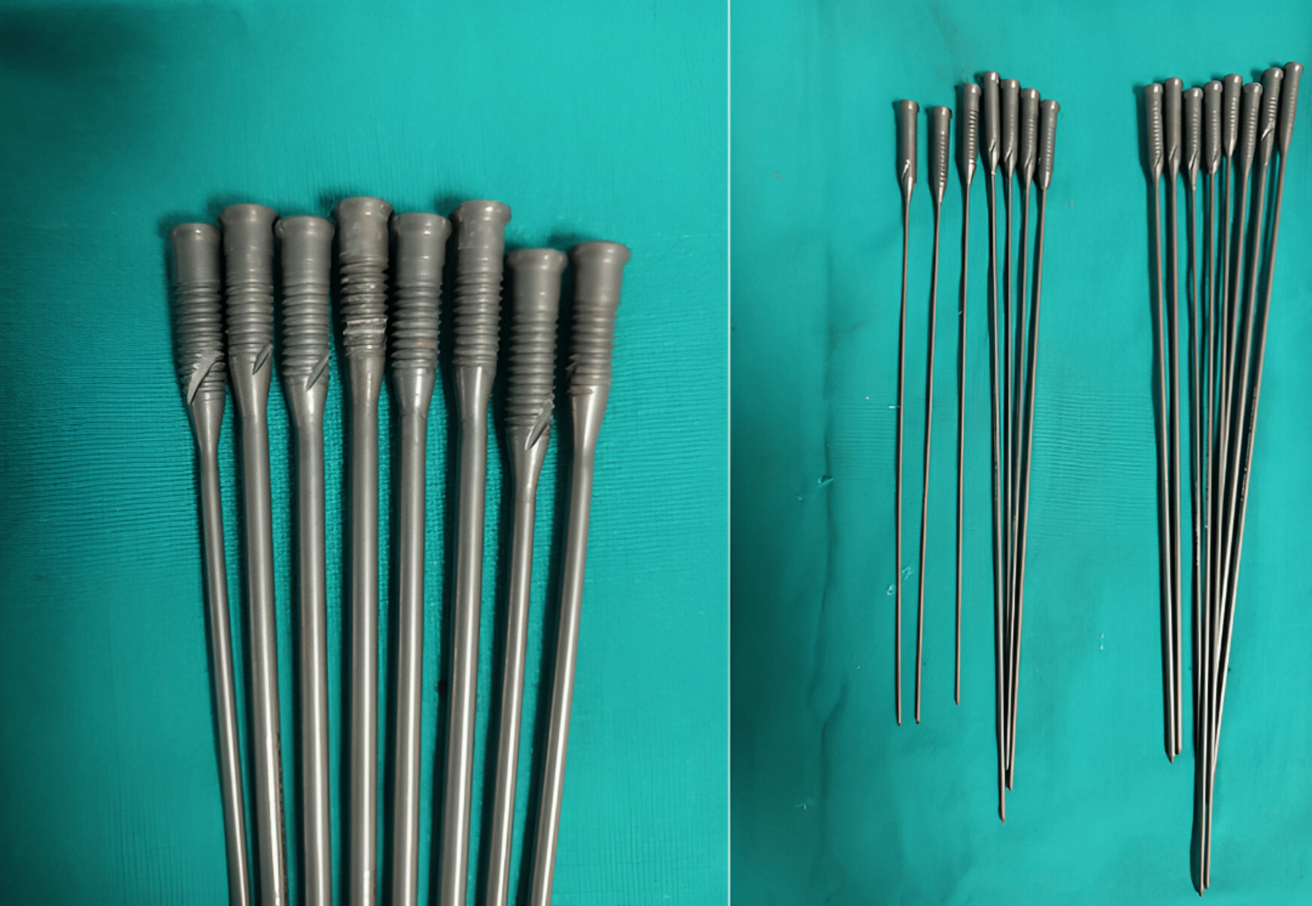
Photograph of the screw nail showing the threaded proximal end, which engages the cancellous region of the lateral end of the clavicle.

The titanium screw nail is a flexible, smooth, circular implant available in diameters of 2 mm, 3 mm, and 4 mm, featuring a beveled tip. Its design includes a threaded head integrated with the nail, which is secured in place by a circular running notch at the end of the nail shaft. This configuration enables the self-cutting thread to be advanced and fixed using a 3.5 mm screwdriver. The threaded end has adequate purchase in the lateral cancellous portion of the clavicle and reduces the risk of backout. The beveled distal end facilitates fracture reduction and aids in engaging the subchondral bone, enhancing stability, as shown in Figure 1.

Surgical procedure

**Figure 2:**
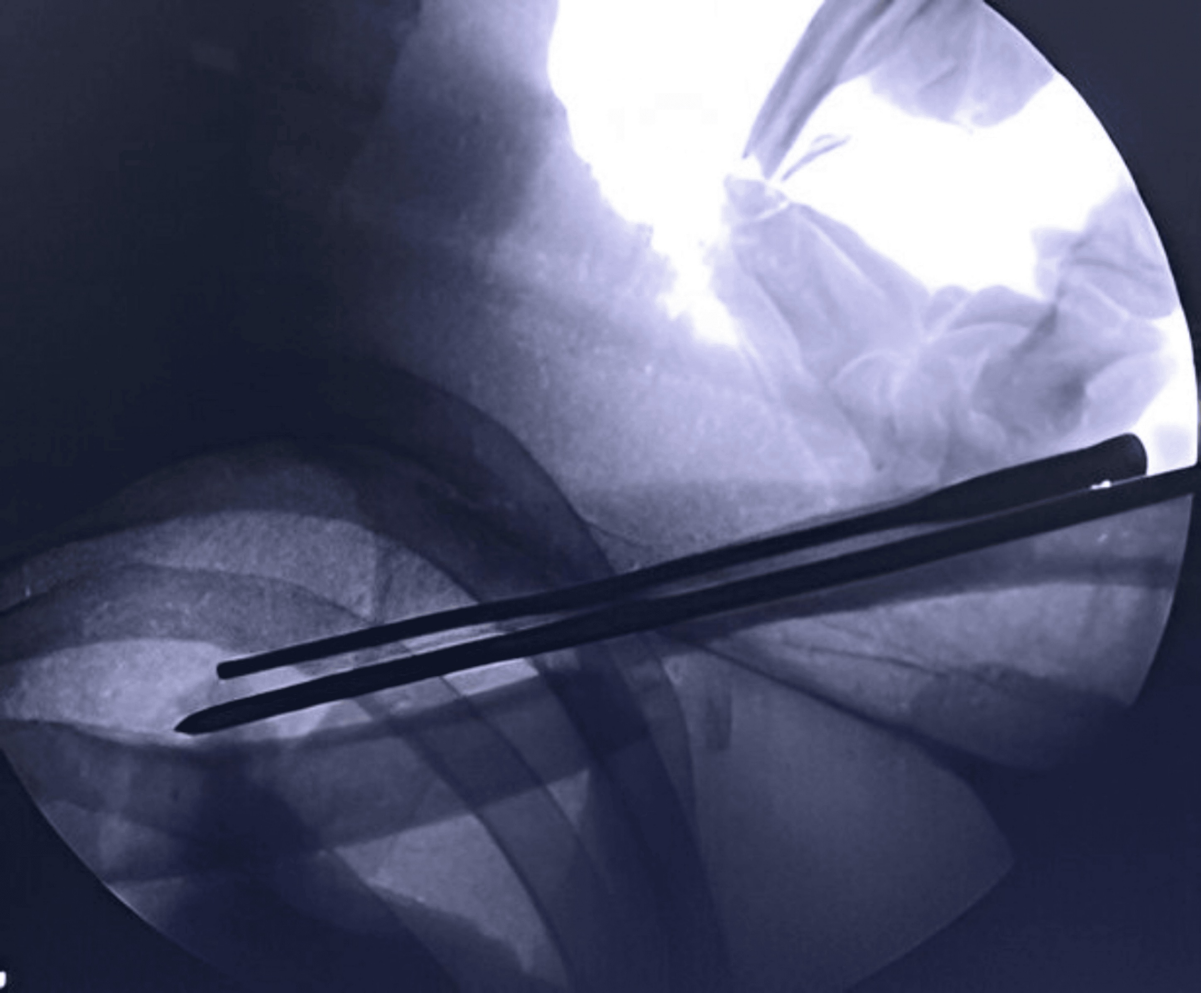
Intraoperative fluoroscopy image confirming the screw length.

The patients were operated on a radiolucent table and underwent the procedure under general anesthesia. The size of the screw nail was measured under fluoroscopy (Figure 2).

**Figure 3:**
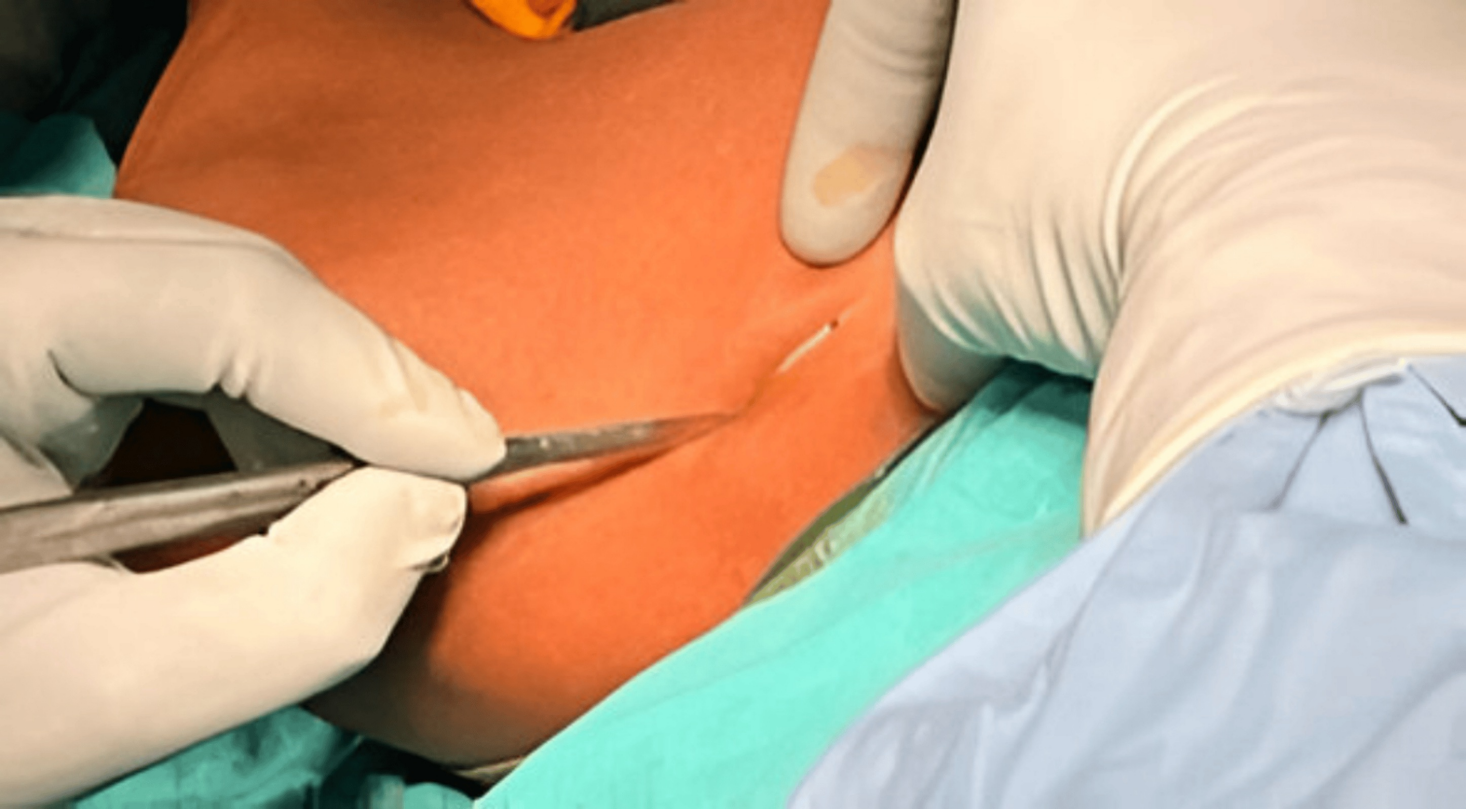
Incision over the fracture site under fluoroscopic guidance.

Under fluoroscopic guidance, a small incision measuring 1 to 1.5 cm was made over the fracture site (Figure 3).

**Figure 4:**
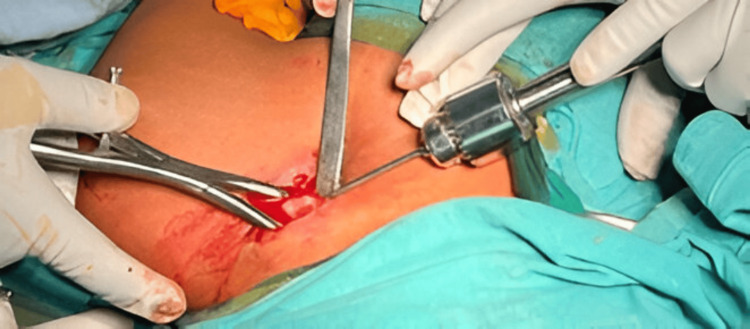
K-wire passed through the lateral fracture fragment.

The medial and lateral ends of the fracture site were exposed, and any soft tissue interposed at the fracture site that would prevent reduction was cleared. A sharp-pointed 3 mm Kirschner wire was inserted intramedullary through the lateral fracture fragment with the help of a T-handle (Figure 4).

**Figure 5:**
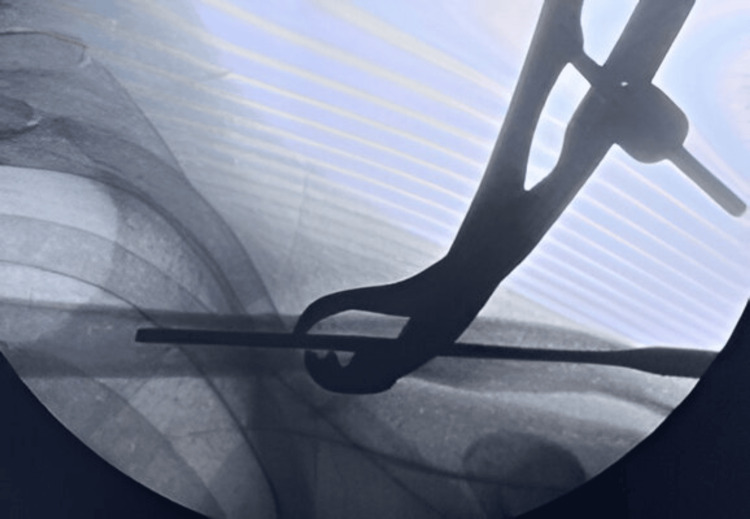
Fracture reduction under fluoroscopic guidance and advancement of the screw nail into the medial cortex.

The Kirschner wire was advanced laterally, and a lateral entry for the screw nail was created by the sharp tip at the posterolateral end of the clavicle. The Kirschner wire was further advanced to make a stab incision over the skin on the posterolateral aspect of the shoulder to create the entry for the screw nail. The screw nail was inserted from the lateral entry point of the clavicle with the help of a screwdriver and advanced through the fracture site, which was reduced under direct vision; the screw nail was then advanced into the medial fracture fragment (Figure 5).

**Figure 6:**
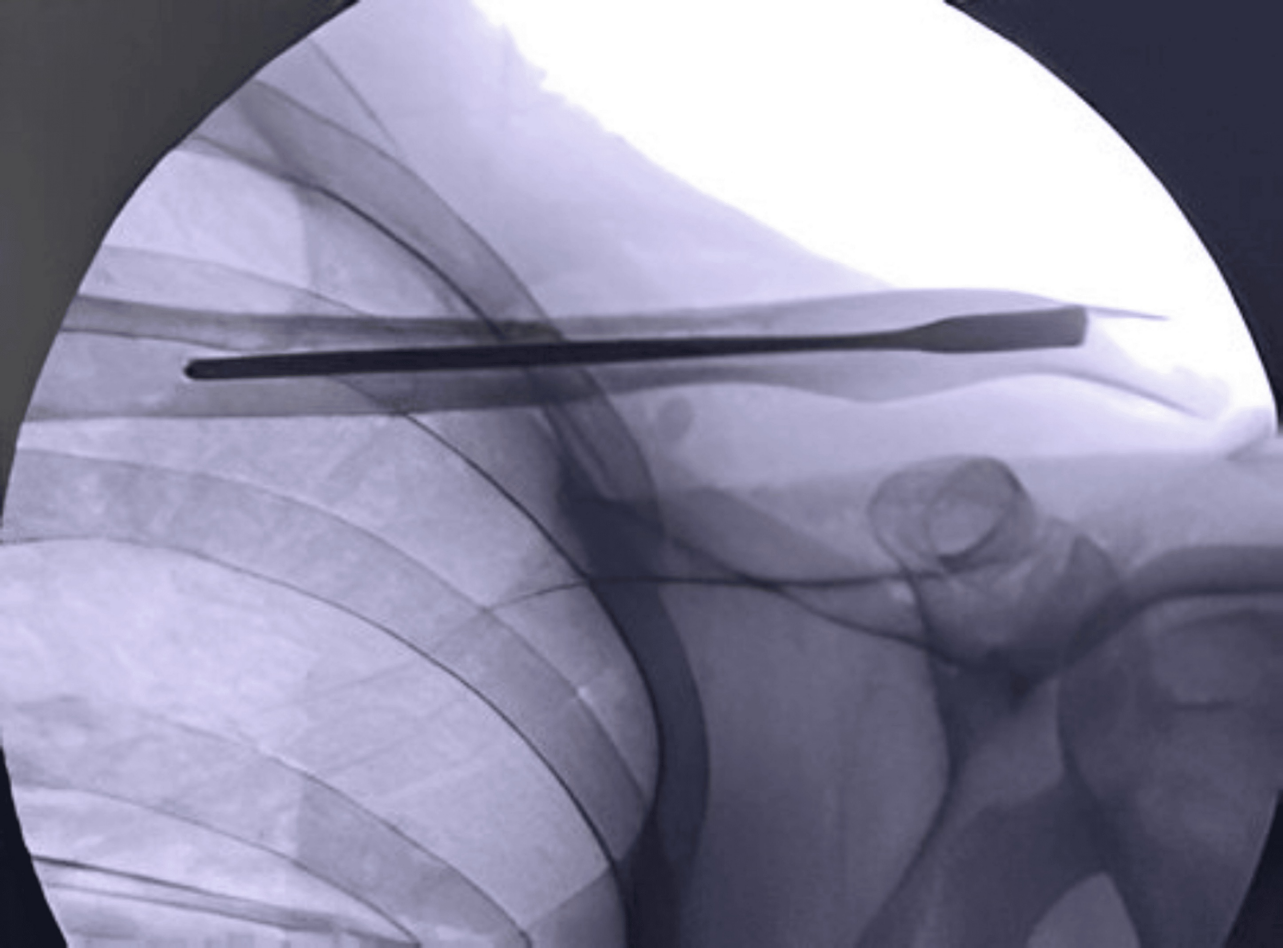
Final fluoroscopy image after screw nail insertion.

The threads at the lateral end of the screw nail are self-tapping and engage the lateral metaphyseal end of the clavicle to avoid backout. One-fourth of the threaded end of the screw nail was purposefully kept outside the cortex for convenient implant removal in the future. Throughout the procedure, fluoroscopy was used to assess fracture reduction and implant positioning (Figure 6).

Once confirmed under fluoroscopy, the incision was closed with sutures. Postoperative X-ray imaging was routinely performed to evaluate fracture reduction and screw placement.

All patients received standard preoperative and postoperative care to ensure consistent treatment outcomes.

Postoperative protocol

Postoperatively, all patients were immobilized in an arm sling for four weeks. Pendulum exercises were initiated at two weeks, and active-assisted shoulder movements were started after four weeks. Full range of motion and strengthening exercises were gradually introduced thereafter.

Evaluation parameters

Patients were evaluated clinically and radiographically at 3, 6, and 12 months postoperatively. Parameters recorded included: operative time, incision length, and intraoperative blood loss; duration of hospital stay; radiological union time; functional outcome measured using the DASH score; and complications such as infection, implant backout, delayed union, or neurovascular injury.

Statistical analysis

All data were entered in Microsoft Excel and analyzed using the SPSS software, version 25.0 (IBM Corp., Armonk, NY, USA). Continuous variables were expressed as mean ± SD, and categorical variables as frequencies and percentages. The paired t-test was used to compare preoperative and postoperative DASH scores. A p-value < 0.05 was considered statistically significant. A post-hoc power analysis confirmed that the sample provided >80% power to detect a clinically significant difference in functional outcomes.

## Results

Based on the Allman classification, all the patients included had Type 1 clavicle fractures (midshaft fractures). In our study, 18 patients were male and six were female; the mean age of all patients was 35.5 years, ranging from 20 to 76 years. The primary causes of injury included road traffic accidents (15 cases), assaults (4 cases), falls from height (2 cases), and domestic falls (3 cases) (Table 1). Surgical procedures were performed within the first seven days following the injury. The average follow-up period was 12 months. All patients were followed up regularly for clinical and radiological evaluation.

**Table 1:** Patient information.

Patient parameters	Values
Age (years), mean	35.5
Male, n	18
Female, n	6
Mode of injury	
Road traffic accident, n	15
Domestic fall, n	3
Direct assault, n	4
Fall from height, n	2

The average surgical duration, perioperative blood loss, and length of hospital stay were 45.41 minutes, 80.83 milliliters, and 4.91 days, respectively. The mean incision length at the fracture site was 13.91 mm. One patient had delayed union (Table 2).

**Table 2:** Assessment parameters.

Parameters	Values
Blood loss (mL)	80.83
Incision length (mm)	13.91
Duration of surgery (minutes)	45.41
Duration of hospital stay (days)	4.91
DASH score	
Preoperative	73.5
Postoperative (3 months)	59.4
Postoperative (6 months)	43.5
Postoperative (12 months)	18.8
Complications (no. of patients)	
Implant backout	1
Infection	None
Implant breakage	None
Outcome	
Excellent	19
Fair	5
Poor	0

No postoperative complications, such as infection, incision numbness, hypertrophic scarring, nail-end irritation, nail displacement, or breakage, were observed. The titanium screw nails used in the procedures had a diameter of 3 mm.

**Figure 7:**
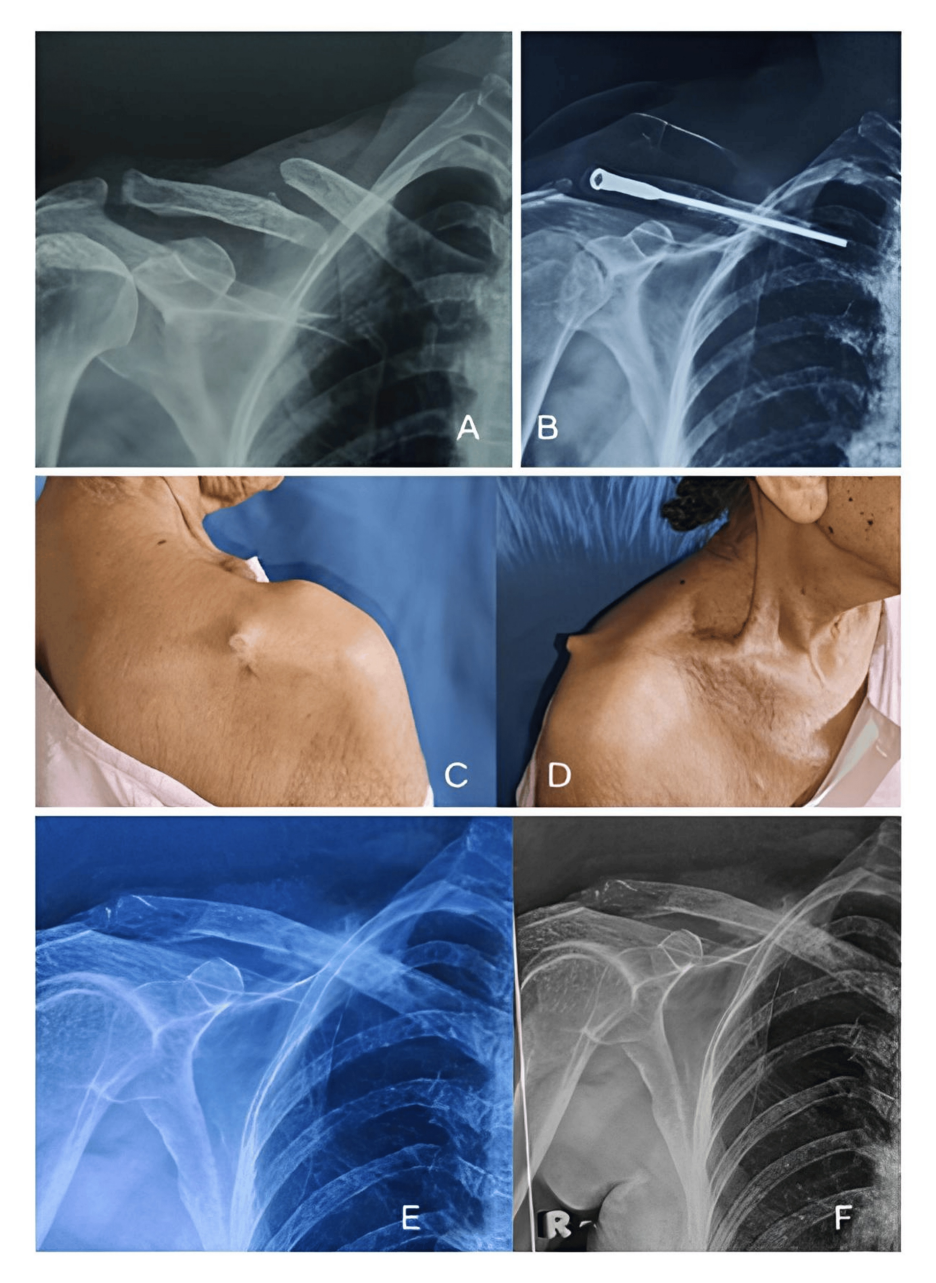
A 76-year-old female with a right middle-third displaced clavicle fracture treated with retrograde screw nailing. (A) Preoperative X-ray; (B) Postoperative X-ray; (C, D) Clinical images showing screw backout from the posterolateral aspect; (E) X-ray after removal of the screw nail; (F) X-ray 6 months after removal of the screw nail showing complete union of the clavicle fracture.

Postoperatively, one elderly patient with osteoporosis reported pain in the posterolateral aspect of the shoulder region due to backout of the nail head from the lateral cortex four months after surgery, for which the nail was removed. There were clinical signs of bony union and radiological signs of soft callus formation. Shoulder movements were started gradually after implant removal. Fracture healing in this patient was achieved in 10 weeks. Eventually, the patient had an excellent functional outcome with no other complications (Figure 7).

During postoperative follow-ups, none of the patients demonstrated signs of failure of reduction or increased displacement. All patients reported satisfaction with their outcomes.

**Figure 8:**
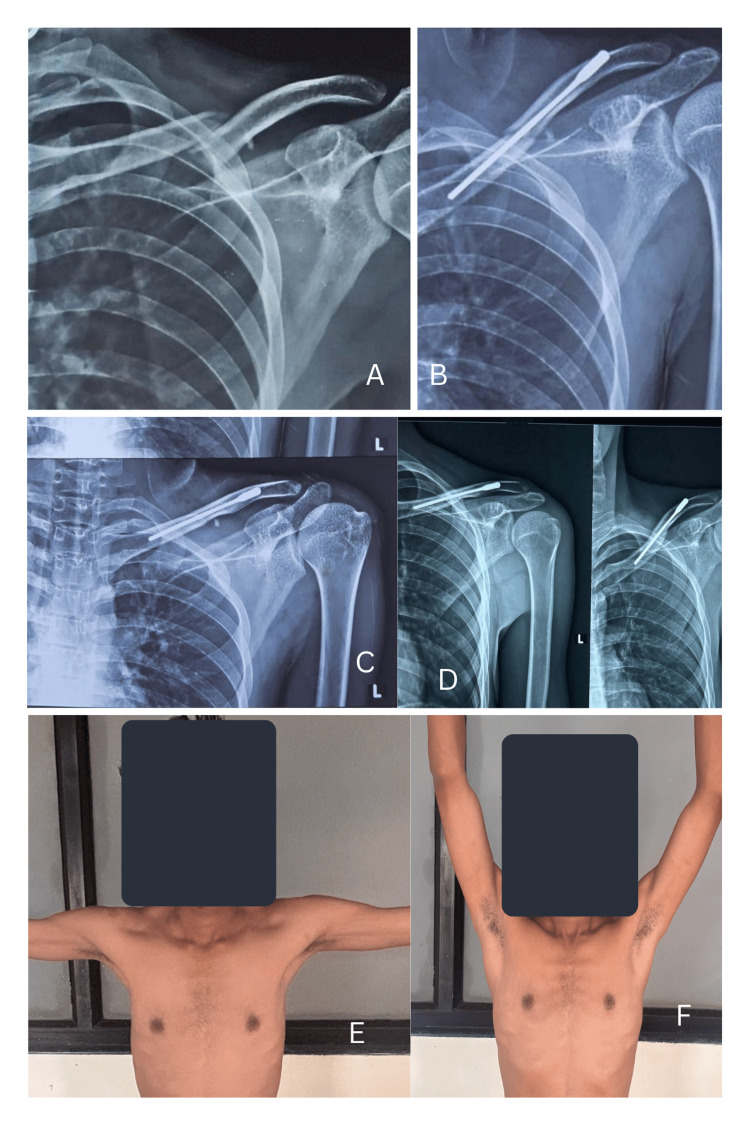
A 30-year-old male patient with a middle-third clavicle fracture treated with a screw nail. (A) Preoperative radiograph showing an Allman Type 1 displaced clavicle fracture; (B) Immediate postoperative radiograph; (C) Radiograph at 6 months; (D) Radiograph at 1 year postoperatively; (E, F) Clinical images showing complete shoulder range of motion.

Three days post-surgery, functional outcomes showed significant improvement. Additionally, shoulder abduction improved notably within two weeks postoperatively. At the final follow-up, the average DASH score was 18.8 (Table 2), with 79.2% of patients achieving excellent results and 20.8% obtaining good results. A representative case is illustrated in Figure 8.

## Discussion

When compared to plate fixation, intramedullary fixation, particularly TEN nailing, has demonstrated superior clinical outcomes in treating displaced midshaft clavicular fractures. These benefits include reduced operative time, smaller surgical wounds, faster subjective pain relief, and improved postoperative shoulder joint function. Additionally, TEN has been associated with a shorter average bone healing period, quicker functional recovery, greater patient satisfaction, and higher approval regarding cosmetic appearance [17].

Traditional TENs are primarily stabilized by their arc-shaped nail heads, which results in limited resistance to rotational and axial displacement. Furthermore, the clavicle’s unique anatomical S-shaped curvature, irregular structure, and narrow medullary cavity pose challenges for TEN insertion. The larger diameter of standard TENs can make insertion difficult and may even lead to fractures at the distal end. For midshaft clavicular fractures, commonly used TENs have an average diameter of 2 mm. However, intramedullary fixation with conventional TENs is frequently associated with postoperative complications. These may include nail migration, skin irritation, lateral cortex perforation, and, in some cases, fracture-site shortening or displacement [18].

Our study demonstrated excellent functional recovery and a 100% union rate, with one case of delayed union. These findings align with those of Andrade-Silva FB et al. (2015), who reported a 97% union rate using TEN and highlighted minimal soft-tissue disruption compared with plating [19]. Similarly, Frigg A et al. (2009) found that intramedullary fixation offered shorter operative time, smaller incisions, and superior cosmetic outcomes while maintaining adequate stability [20]. In our study, the mean operative time was 45.4 minutes and the mean incision length was 13.9 mm, further confirming the minimally invasive advantage of the intramedullary approach.

In comparison, van der Meijden OA et al. (2012) and the Canadian Orthopaedic Trauma Society (COTS, 2007) reported that plate fixation, though biomechanically stronger, was associated with increased soft-tissue irritation and higher rates of reoperation for implant removal [21-22]. Our results suggest that retrograde screw nailing offers comparable union rates with fewer implant-related complications and better patient satisfaction. No cases of infection, implant failure, or neurovascular injury occurred in our series.

The improvement in the Disabilities of the Arm, Shoulder and Hand (DASH) score from 73.5 preoperatively to 18.8 at 12 months (p < 0.001) demonstrates significant functional recovery. This is consistent with findings by Chen Y et al. (2018), who reported similar postoperative DASH improvements following intramedullary fixation. The stable intramedullary construct likely contributes to early mobilization and a faster return to daily activities [23].

The retrograde entry technique used in this study offers several advantages over the traditional antegrade approach. It avoids disruption of the medial clavicular region and reduces the risk of implant migration. The lateral entry also facilitates screw removal under local anesthesia, if needed.

Limitations

This study has certain limitations, including a relatively small sample size, the absence of a control group, and a limited follow-up duration of one year. Moreover, the study did not include a cost-effectiveness analysis or a patient-reported cosmetic assessment, which could add value to the analysis.

Future directions

Larger, multicenter randomized controlled trials comparing retrograde screw nailing with plate fixation and other intramedullary devices are warranted to confirm these findings and establish standardized guidelines for surgical management.

## Conclusions

Intramedullary fixation using the newly designed titanium screw nail appears to be a safe and effective approach for treating displaced middle-third clavicle fractures, offering minimal complications and favorable clinical outcomes.
